# Combined hyperbaric oxygen and repetitive transcranial magnetic stimulation for vascular cognitive impairment: a randomized controlled trial

**DOI:** 10.3389/fneur.2026.1826010

**Published:** 2026-08-18

**Authors:** Yixiu Lin, Xinxin Chen, Dan Li, Jiao Luo, Lin He, Yi Chen, Cheng Huang

**Affiliations:** 1 Department of Rehabilitation Medicine, Center for High Altitude Medicine, West China Hospital, Sichuan University, Chengdu, Sichuan, China; 2 Department of Rehabilitation Medicine, The First Affiliated Hospital, Sun Yat-sen University, Guangzhou, Guangdong, China; 3 West China School of Nursing, Sichuan University, Chengdu, Sichuan, China; 4 Key Laboratory of Rehabilitation Medicine in Sichuan Province, West China Hospital, Sichuan University, Chengdu, Sichuan, China

**Keywords:** functional near infrared spectroscopy, hyperbaric oxygen therapy, randomized controlled trial, repetitive transcranial magnetic stimulation, vascular cognitive impairment

## Abstract

**Background:**

Effective rehabilitation strategies for vascular cognitive impairment (VCI) still remain limited. This study was aimed to explore the clinical efficacy and related mechanisms of hyperbaric oxygen therapy (HBOT) combined with repetitive transcranial magnetic stimulation (rTMS) for VCI.

**Methods:**

72 participants were recruited and randomly assigned to the control, HBOT, rTMS, and Combined (HBOT combined with rTMS) groups. The primary outcome measures were the Montreal Cognitive Assessment (MoCA) and the Mini-Mental State Examination (MMSE), while the secondary outcome measures comprised the Modified Barthel Index (MBI), the Self-rating Anxiety Scale (SAS), and the Self-Rating Depression Scale (SDS). The concentration of oxygenated hemoglobin (HbO_2_) in the cerebral cortex was measured by near-infrared spectroscopy (fNIRS). These indicators were measured at the baseline (T1) and 3 weeks after the intervention (T2).

**Results:**

After treatment, the significant improvements in MoCA, MMSE, and MBI scores, and the reductions in SAS and SDS scores were observed across all groups (all *p* < 0.05). Regarding the primary outcomes, the combined group showed significantly greater improvement in MoCA and MMSE scores compared to each of the other three groups (*p* < all 0.05). For the secondary outcomes, the combined treatment group showed significantly greater improvement in MBI scores only than the control group (*p* < 0.05), and no significant inter-group differences were observed in the changes of SAS or SDS scores before and after treatment (all *p* > 0.05). Additionally, fNIRS analysis following FDR correction showed that CH9 and CH45 channels had statistically significant differences in HbO_2_ after treatment (all *p* < 0.05), though a sensitivity analysis suggested these findings may be partially influenced by physiological noise.

**Conclusion:**

Combined HBOT and rTMS therapy demonstrates superior efficacy in improving cognitive function which may be mediated by the primary somatosensory cortex and primary motor cortex of the brain. However, the combined regimen did not yield greater improvement in daily function or mood. Combined HBOT + rTMS is a promising cognitive rehabilitation strategy in the treatment of VCI.

**Clinical trial registration:**

https://www.chictr.org.cn/showproj.html?proj=190934, Identifier ChiCTR2300068242.

## 1 Introduction

Vascular cognitive impairment (VCI) is increasingly prevalent in the aging population, driven primarily by vascular risk factors and cerebrovascular diseases (1). Its rising incidence, prevalence, disability rate, and mortality impose a significant socioeconomic burden (1). Patients with VCI typically exhibit impairments in at least one cognitive domain, such as executive function, attention, memory, or language etc., and may also experience behavioral and psychological symptoms like apathy, anxiety, and depression (2). Post-stroke VCI is particularly common, and cognitive dysfunction is closely associated with poor patient prognosis (3). Without timely intervention, VCI may progress to vascular dementia (VaD), which accounts for 15–30% of all dementia cases (4). Etiological therapies for VCI remain lacking, and pharmacological options are limited by frequent contraindications and modest efficacy. This highlights the need for alternative therapeutic strategies.

Hyperbaric oxygen therapy (HBOT) involves breathing high-concentration oxygen (100%) in an environment exceeding 1 atmosphere absolute (ATA) (5). A meta-analysis confirmed that HBOT significantly improves cognitive function and activities of daily living in both Alzheimer’s disease (AD) and VaD (6). Xu et al. (7) found that after 12 weeks of HBOT (2.0 ATA), VaD patients showed improved cognitive function, elevated serum Humanin levels, and enhanced cerebral metabolism. Preclinical studies suggest that the neuroprotective effects of HBOT are mediated through multiple mechanisms relevant to VCI, including increased cerebral perfusion, suppression of hippocampal neuron apoptosis, and attenuation of neuroinflammation and oxidative stress (8–11). The mechanisms of HBOT for VaD also involve elevating cerebral oxygen partial pressure, promoting angiogenesis and neurorepair, as well as reducing oxidative stress and enhancing mitochondrial function (7, 8, 11, 12). These findings position HBOT as a promising candidate for VCI rehabilitation.

Repetitive transcranial magnetic stimulation (rTMS) is a non-invasive neuromodulation technique that uses pulsed magnetic fields to regulate the excitability of cortical neurons (13). A meta-analysis indicated that rTMS has positive effects on attention, memory, working memory, and global cognition in patients with post-stroke cognitive impairment (PSCI) (14). Yin et al. (15) demonstrated that 10 Hz rTMS not only enhanced cognitive and daily living function but also modulated functional connectivity within task relevant networks. Basic studies suggested that the mechanisms of rTMS for VCI may involve enhancing neuronal excitability, promoting synaptic plasticity, upregulating the expression of brain-derived neurotrophic factor (BDNF) and vascular endothelial growth factor, restoring cholinergic system activity, and modulating apoptosis-related proteins (16–19).

Functional near-infrared spectroscopy (fNIRS) is a non-invasive brain imaging technology that can detect real-time changes in oxygenated hemoglobin (HbO₂) and deoxygenated hemoglobin (HbR) concentrations in the cerebral cortex, reflecting cortical activation during cognitive tasks (20, 21). An increase in HbO₂ often indicates enhanced cortical excitability and serves as an important indicator for assessing cognitive function (22). Studies have successfully employed fNIRS to map rTMS-induced cortical excitability changes, establishing it as a practical tool for probing treatment mechanisms in neuromodulation trials (23, 24).

Although most studies supported the cognitive benefits of HBOT and rTMS, some reports indicated uncertain efficacy (25–28). Therefore, this study aimed to further validate the cognitive improvement effects of these two interventions in VCI. Given their distinct mechanisms of action, we hypothesized that both HBOT and rTMS, either alone or in combination, would produce clinically meaningful improvements in cognitive function, and that combination therapy might yield synergistic effects. Furthermore, this study used fNIRS to monitor changes in cortical HbO₂ concentration during cognitive tasks before and after treatment, in order to explore the potential neural mechanisms underlying HBOT and rTMS induced cognitive improvement in VCI.

## 2 Materials and methods

This clinical trial is a prospective, assessor-blind, randomized controlled trial, conducted in accordance with the CONSORT criteria. The study was approved by the Biomedical Ethics Review Committee of West China Hospital, Sichuan University [ethical referenced number: 2022 (1972)]. The clinical trial registration number is ChiCTR2300068242. Written informed consent was obtained from all participants.

### 2.1 Participants

VCI is a broad term that encompasses both mild vascular VCI and VaD according to the Chinese Guidelines for Diagnosis and Treatment of vascular cognitive impairment (V.2019) (29). Both subtypes were eligible for this study. The diagnosis of VCI was based on three core elements from the guidelines (29): (1) objective cognitive impairment in at least one domain (executive function, attention, memory, language, or visuospatial abilities) confirmed by neuropsychological testing; (2) evidence of cerebrovascular disease, including vascular risk factors, stroke history, or neuroimaging findings (CT or MRI) showing vascular brain lesions such as large vessel infarction, lacunar infarcts, or extensive white matter hyperintensities; and (3) determination that cerebrovascular pathology is the predominant cause of the cognitive impairment, with exclusion of other causes such as Alzheimer’s disease. Patients who met these diagnostic criteria were screened for enrollment according to the following inclusion criteria: (1) Age 20–80 years; (2) Mini-Mental State Examination (MMSE) score <25 or Montreal Cognitive Assessment (MoCA) score <26; (3) Educational attainment at primary level or above; (4) Signed informed consent provided. The exclusion criteria were as follows: (1) History of other neurological disorders (e.g., brain tumor, encephalitis, or Alzheimer’s disease); (2) Unstable or abnormal vital signs, or rapidly progressing illness; (3) Severe impairment of speech, hearing, or vision; (4) Unconsciousness or persistent vegetative state; (5) Severe mental disorder or significant depression; (6) History or family history of epilepsy; (7) Severe cardiac or pulmonary dysfunction (e.g., dyspnea, hypoxia, cyanosis); (8) rTMS contraindications (e.g., intracranial metal implants, implanted electronic devices, or pregnancy); (9) HBOT contraindications (e.g., pneumothorax, claustrophobia, pulmonary bullae, or severe infection). The causes of VCI in the enrolled participants were ischemic stroke and hemorrhagic stroke, confirmed by CT or MRI.

### 2.2 Study design and settings

All participants were randomly assigned to one of four groups: control group, HBOT group, rTMS group, or HBOT combined with rTMS group. A computer-generated randomization sequence (using SPSS software) was prepared by an independent statistician, with allocation concealed in opaque, sequentially numbered, sealed envelopes. All interventions were administered once daily, 5 days per week, for 3 weeks. All enrolled participants received conventional treatment.

### 2.3 Interventions

All enrolled participants received conventional treatment (CT), including pharmacotherapy for cerebrovascular diseases, cognitive training, exercise therapy, physical factor therapy, occupational therapy, and traditional Chinese medicine therapy. No participant in any group received memantine or other NMDA receptor antagonists during the study period. Participants in the control group received CT only. Those in the HBOT group received HBOT in addition to CT; participants in the rTMS group received rTMS in addition to CT; and participants in the combination group received both HBOT and rTMS alongside CT. For participants in the combined treatment group, rTMS was delivered first, followed by a 30-min rest interval before HBOT.

#### 2.3.1 HBOT

HBOT was administered using a 3-compartment, 7-door medical hyperbaric oxygen chamber (manufactured by Yantai Binglun Hyperbaric Oxygen Chamber Co., Ltd., Shandong Province, China). The treatment pressure was set at 2.0 ATA. The compression time was 20 min, and the decompression time was 20 min. When the chamber pressure stabilized, patients breathed 100% oxygen via a facemask for 60 min, with a 10-min break in between to breathing air. Each HBOT session lasted 110 min, administered once daily, 5 days per week, for a total of 3 consecutive weeks.

#### 2.3.2 rTMS

rTMS treatment was delivered using a magnetic stimulator with a figure-8-shaped coil (YRD CCY-I, YIRUIDE Medical Equipment Co., Ltd., Wuhan, China). The left dorsolateral prefrontal cortex (DLPFC) was localized using the international 10–20 electroencephalography system (30). The vertex (Cz) was first identified as the intersection of the nasion–inion midline and the line connecting the left and right preauricular points. The F3 electrode position, corresponding to the left DLPFC, was then marked at 20% of the distance from Cz to the left preauricular point along the Cz–left preauricular line. The rTMS coil was placed tangentially over the F3 location (31). The stimulation intensity was set at 80% of the motor threshold. The motor threshold level was defined as the lowest stimulation intensity required to elicit motor evoked potentials (greater than 50 μV) in at least 5 out of 10 trials from the right abductor pollicis brevis muscle (32). The rTMS protocol used a stimulation frequency of 10 Hz (16, 33)(5 s durations, 25 s pause), delivering a total of 2000 pulses per session. Each rTMS session lasted 20 min, administered once daily, 5 days per week, for a total of 3 consecutive weeks.

### 2.4 Outcome indicators

Baseline information of the subjects was collected, and all outcome measures were assessed by a single therapist blinded to group allocation before and after the three-week intervention.

#### 2.4.1 Cognitive function

The Montreal Cognitive Assessment (MoCA) scale was a rapid screening tool for mild cognitive impairment, offering higher sensitivity but lower specificity compared to the MMSE (34). The Beijing version of the MoCA (MoCA-BJ) was used in this study, which has been well-validated in Chinese populations (35). It assessed visuospatial abilities, executive functions, verbal fluency, orientation, naming, delayed recall, attention, abstraction, and calculation. The score ranged from 0 to 30, with a score <26 suggesting cognitive impairment. One point was added to the total score for subjects with ≤12 years of education. The MoCA was administered at baseline (T1) and after 3 weeks of intervention (T2). Individual item scores and total scores were recorded. The Mini-Mental State Examination (MMSE) scale was a commonly used and effective tool for screening cognitive function in stroke patients (36). It consisted of 30 items across five domains: memory, attention and calculation, language and recall, and orientation. Each item was worth 1 point, totaling 0–30. A higher score indicated better cognitive ability. A total MMSE score ≤26 indicated cognitive impairment. The MMSE was administered at T1 and T2, and the total score was recorded.

#### 2.4.2 Activities of daily living

The modified Barthel index (MBI) scale was used to measure the participants’ performance in activities of daily living and had high reliability (37). It assessed ten items: Grooming, feeding, dressing, toilet use, bathing, bed-chair transfer, ambulation/wheelchair use, stair climbing, bladder control, and bowel control. The score ranged from 0 to 100, with a higher score indicating better functional independence. Scores were interpreted as: 0–25 (total dependence), 25–49 (severe dependence), 50–74 (moderate dependence), 75–90 (mild dependence), 91–99 (minimal dependence), and 100 (complete independence). The MBI was administered at T1 and T2. Individual item scores and the total score were recorded.

#### 2.4.3 Assessment of anxiety and depression

The Self-Rating Anxiety Scale (SAS) was used to assess the severity of anxiety symptoms (38). It consisted of 20 items rated on a 4-point scale (“occasionally or none,” “sometimes,” “often,” “always”). The raw score sum was multiplied by 1.25 to obtain a standard score ranging from 25 to 100. A standard score ≥50 suggested clinical anxiety, categorized as mild (39–48), moderate (49–58), or severe (>69). The scale’s Cronbach’s *α* coefficient was 0.82. The SAS was administered at T1 and T2, and the total score was recorded. The Self-Rating Depression Scale (SDS) was used to assess the severity of depressive symptoms (59). Its structure and scoring were identical to the SAS. A standard score ≥53 suggested clinical depression, categorized as mild (42–51), moderate (52–58, 60–62), or severe (>72). Its Cronbach’s *α* coefficient was 0.84. The SDS was administered at T1 and T2, and the total score was recorded.

#### 2.4.4 HBO₂ concentration of cerebral cortex

**Figure 1:**
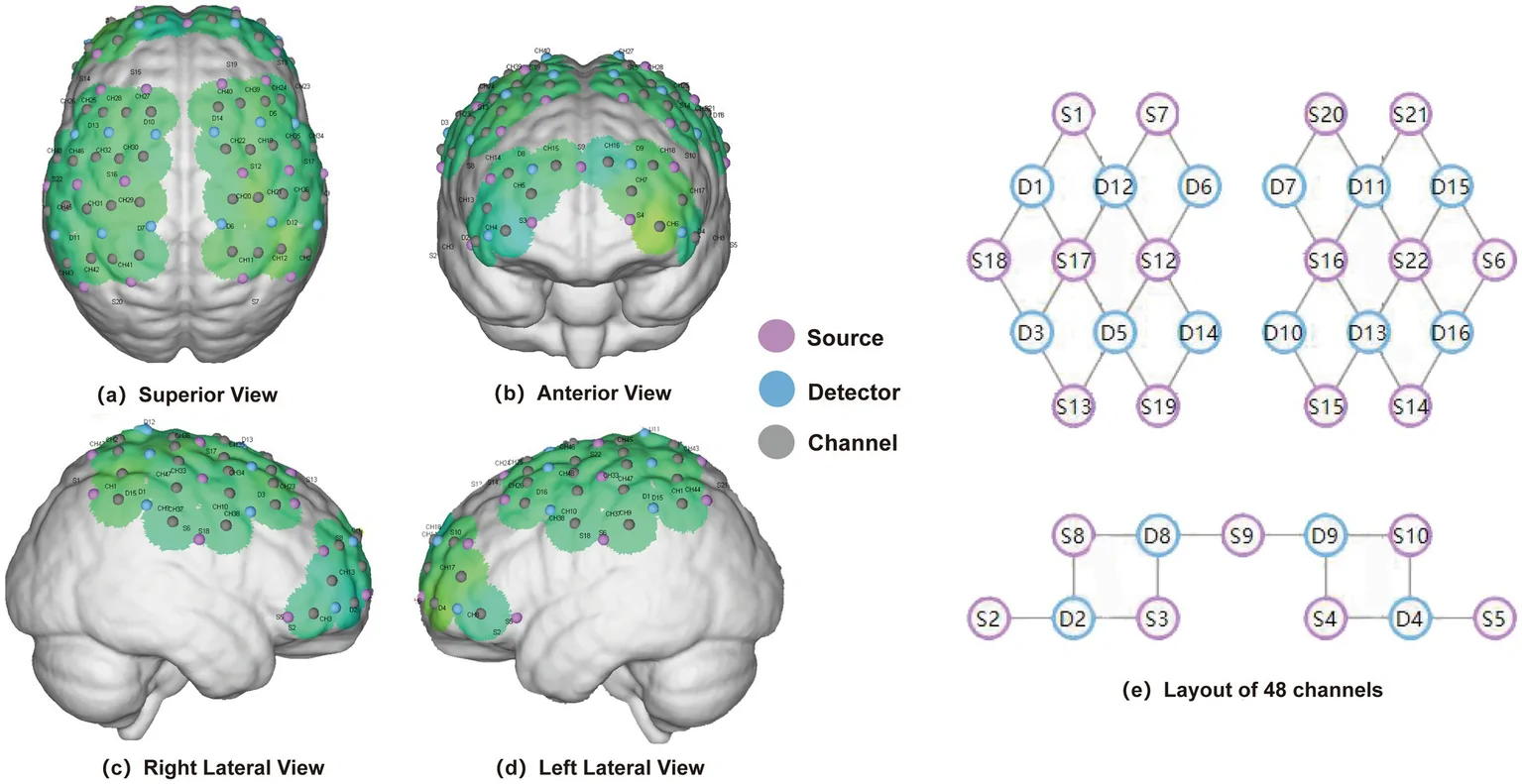
Optode layout and channel configuration. **(a)** Superior View. **(b)** Anterior View. **(c)** Right Lateral View. **(d)** Left Lateral View. **(e)** Layout of 48 channels.

Changes in cerebral blood flow during task execution were measured using a multi-channel fNIRS system (NirSmart-6000A, HuiChuang Medical Equipment Co., Ltd., Danyang, China) with dual wavelengths (730 nm and 850 nm). The full-channel sampling frequency was ≥11 Hz. The acquisition cap consisted of 16 detectors and 22 sources, forming 48 effective channels. The average source-detector separation was 30 mm. Placement followed the international 10–20 system, covering frontal and parietal regions. Figure 1 showed the source and detector positions. Table 1 showed the correspondence between fNIRS channels and Brodmann areas. A working memory (WM) task served as the cognitive test. WM involves the temporary storage and manipulation of information. One WM test block sequentially included 10 s rest, 20 s WM search task, 10 s rest, 20 s non-WM search task, and 10 s rest (total 70 s). In the WM search task, three figures appeared sequentially on a tablet. Subjects were required to remember their shapes, colors, and order, and were then asked to identify and tap them in sequence from eight figures. The non-WM search task required tapping figures in the presented order without memorization. Upon entering the assessment room, subjects were allowed to acclimate to the environment. After donning the fNIRS cap, subjects performed the WM test (repeated four times consecutively) on the tablet while cerebral hemodynamics were recorded (total 280 s). fNIRS data acquisition and preprocessing followed the best practice guidelines for fNIRS publications (63). The collected fNIRS data were analyzed using the NirSpark software package (HuiChuang Medical Equipment Co., Ltd., China).

**Table 1:** The correspondence between fNIRS channels and the Brodmann area.

Channel	Brodmann area	Channel	Brodmann area
CH1 (S1–D1)	39, 40	CH25 (S14–D13)	6, 8, 9
CH2 (S1–D12)	2, 39, 40	CH26 (S14-D16)	6, 9, 44
CH3 (S2–D2)	45, 46, 47	CH27 (S15–D10)	6, 8
CH4 (S3–D2)	10, 11, 46, 47	CH28 (S15–D13)	8, 9
CH5 (S3–D8)	10, 11	CH29 (S16–D7)	3, 4, 6
CH6 (S4–D4)	10, 11, 46, 47	CH30 (S16–D10)	6
CH7 (S4–D9)	10, 46	CH31 (S16–D11)	3, 4, 6
CH8 (S5–D4)	38, 45, 46, 47	CH32 (S16–D13)	6
CH9 (S6–D15)	1, 2, 40, 48	CH33 (S17–D1)	1, 2, 3, 4, 40
CH10 (S6–D16)	1, 3, 4, 6, 43	CH34 (S17–D3)	6, 9
CH11 (S7–D6)	1, 3, 5, 7	CH35 (S17–D5)	4, 6, 8, 9
CH12 (S7–D12)	1, 2, 7, 40	CH36 (S17–D12)	1, 3, 4
CH13 (S8–D2)	10, 45, 46	CH37 (S18–D1)	1, 2, 40
CH14 (S8–D8)	10, 46	CH38 (S18–D3)	3, 4, 6, 43
CH15 (S9–D8)	9, 10	CH39 (S19–D5)	8, 9
CH16 (S9–D9)	9, 10	CH40 (S19–D14)	6, 8
CH17 (S10–D4)	45, 46	CH41 (S20–D7)	1, 5, 7
CH18 (S10–D9)	46	CH42 (S20–D11)	1, 2, 7, 40
CH19 (S12–D5)	6, 8	CH43 (S21–D11)	7, 39, 40
CH20 (S12–D6)	4, 6	CH44 (S21–D15)	39, 40
CH21 (S12–D12)	3, 4, 6	CH45 (S22–D11)	1, 2, 3, 4
CH22 (S12–D14)	6	CH46 (S22–D13)	4, 6
CH23 (S13–D3)	9, 44, 45	CH47 (S22–D15)	1, 2, 3, 40
CH24 (S13–D5)	8, 9	CH48 (S22–D16)	3, 4, 6

fNIRS, functional near-infrared spectroscopy; CH, channel; S, source; D, detector. The optode placement followed the international 10–20 system. Brodmann areas were assigned based on the spatial registration of each channel to the Montreal neurological institute brain atlas.

### 2.5 Statistical analysis

Statistical analysis was performed using SPSS software (version 26.0). The significance level was set at *α* = 0.05. The normality of continuous data was assessed using the Kolmogorov–Smirnov test. Based on the normality test results, data were presented as mean ± standard deviation (SD) or as median (interquartile range). For normally distributed continuous data with homogeneity of variance, within-group comparisons were made using the paired-sample *t*-test, and between-group comparisons were made using one-way analysis of variance (ANOVA). Otherwise, the Wilcoxon signed-rank test was used for within-group comparisons, and the Kruskal–Wallis *H* test was used for between-group comparisons. Categorical data were expressed as frequencies and analyzed using Pearson’s chi-square test or Fisher’s exact test. For normally distributed data with homogeneity of variance, *post-hoc* multiple comparisons were performed using the LSD method following one-way ANOVA. For non-normally distributed data, *post-hoc* pairwise comparisons were conducted using Dunn’s test following the Kruskal–Wallis *H* test.

The NirSpark software package (HuiChuang Medical Equipment Co., Ltd., Danyang, China) was used to analyze the fNIRS data. First, the optical intensity was converted into optical density signals. Motion artifacts were then removed using spline interpolation, and physiological noise (e.g., from Mayer waves, respiration, and cardiac activity) was filtered using a 0.01–0.2 Hz band-pass filter. The filtered optical density data were subsequently converted into concentration changes of oxygenated hemoglobin (Delta-HbO_2_) according to the modified Beer–Lambert law. Neuronal activation is typically accompanied by an increase in HbO₂. HbO₂ is considered to have a better signal-to-noise ratio and is the most sensitive and reliable indicator of changes in regional cerebral blood flow (64). Therefore, for further statistical analysis, the average HbO₂ concentration during the WM search task periods (10–30 s) was calculated separately for the pre-intervention and post-intervention assessments, each based on four consecutive task blocks.

### 2.6 Sample size calculation

The sample size calculation was based on the primary outcome of cognitive function, assessed by the MoCA. Based on a meta-analysis of rTMS for VCI (65), which reported a clinically meaningful between-group MoCA difference of approximately 2.5 points exceeding Cohen’s threshold for a large effect (*f* ≥ 0.40) (66), we set the effect size at *f* = 0.45 *a priori* for the sample size calculation. Using G*Power software (version 3.1) for a one-way ANOVA with four groups, a significance level (*α*) of 0.05 (two-tailed), a power (1 − *β*) of 80%, and an effect size *f* of 0.45, the calculation yielded a total sample size of 60 participants. To account for a potential 20% attrition rate, we aimed to recruit 18 participants per group, resulting in a target total enrollment of 72 participants.

## 3 Results

### 3.1 Baseline characteristics of participants

**Figure 2:**
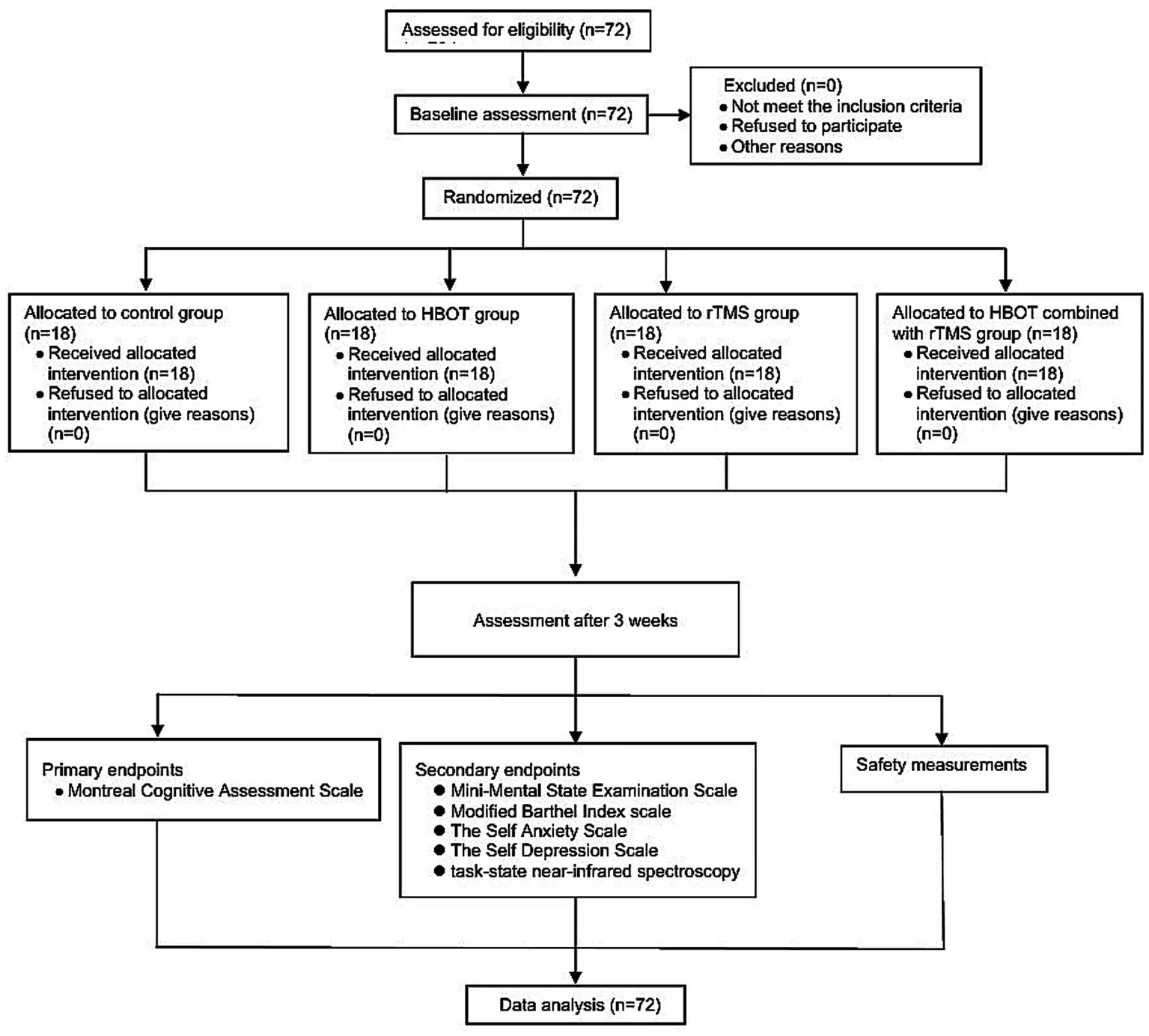
Flow chart of the study.

From November 2021 to December 2023, 72 participants were recruited from the Department of Rehabilitation Medicine at West China Hospital, Sichuan University. All the participants completed the full experiment. Figure 2 illustrated the study flowchart. The mean age of the participants was 54.7 ± 10.9 years. No significant differences were found in any baseline characteristics among the four groups (all *p* > 0.05), as presented in Table 2.

**Table 2:** Baseline characteristics of the participants.

Variables	Control (*n* = 18)	HBOT (*n* = 18)	rTMS (*n* = 18)	HBOT + rTMS (*n* = 18)	*F*/*H*/*χ*^2^	*p*
Age (years)	55.6 ± 8.6	53.8 ± 10.6	53.1 ± 11.3	56.3 ± 13.3	0.339	0.797
Gender (female/male)	11/7	13/5	12/6	14/4	1.309	0.727
Educational level (years)	12 (8)	12 (6)	13.5 (8)	12 (5)	1.652	0.648
Stroke types (infarction/hemorrhage)	11/7	10/8	8/10	11/7	1.350	0.717
Duration (days)	60 (195)	30 (68)	60 (123)	30 (78)	1.639	0.651
Lesion site (left/right/bilateral)	5/11/2	7/6/5	6/10/2	8/8/2	4.560	0.619
Hypertension (yes/no)	12/6	15/3	12/6	10/8	3.258	0.354
Diabetes (yes/no)	4/14	4/14	5/13	5/13	0.421	1.000
Hyperlipidemia (yes/no)	2/16	2/16	2/16	2/16	0.314	1.000
Smoking behavior (yes/no)	1/17	2/16	4/14	2/16	2.220	0.604
MoCA	16.9 ± 4.9	16.9 ± 4.7	16.2 ± 4.6	16.4 ± 4.4	0.098	0.961
MMSE	21.1 ± 3.8	21.6 ± 3.4	20.6 ± 4.6	20.7 ± 4.6	0.204	0.893
MBI	54.3 ± 27.8	54.3 ± 24.8	53.7 ± 19.5	54.9 ± 21.3	0.007	0.999
SAS	32 (12)	33.5 (7)	33 (16)	33 (10)	1.924	0.588
SDS	38.2 ± 11.9	41.4 ± 9.6	39.2 ± 10.2	41.3 ± 9.0	0.441	0.725

HBOT, hyperbaric oxygen therapy; rTMS, repetitive transcranial magnetic stimulation; MoCA, Montreal Cognitive Assessment; MMSE, Mini-Mental State Examination; MBI, modified barthel index; SAS, self-rating anxiety scale; SDS, Self-Rating Depression Scale. Data were presented as mean ± standard deviation for normally distributed continuous variables and median (interquartile range) for non-normally distributed variables. Categorical variables are expressed as frequency (*n*). Between-group comparisons were performed using one-way ANOVA for normally distributed continuous variables, Kruskal–Wallis *H* test for non-normally distributed continuous variables, and chi-square test or Fisher’s exact test for categorical variables. The *F*, *H*, and *χ*^2^ values correspond to the respective test statistics.

### 3.2 Cognitive function

**Figure 3:**
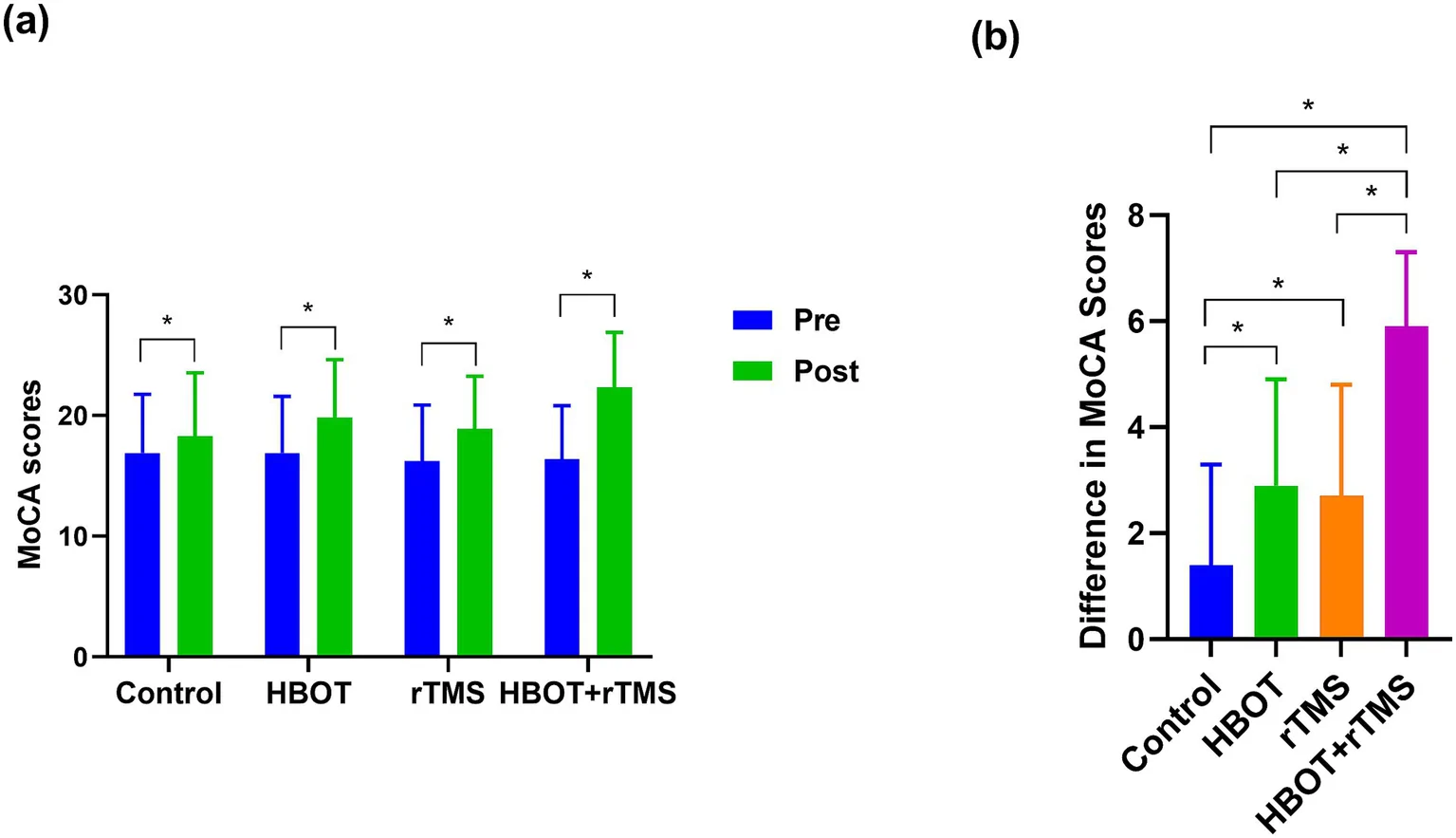
Comparison of MoCA scores before and after treatment in the four groups. **(a)** Within-group comparison of MoCA scores in each group. **(b)** Multiple comparisons of the change in total MoCA scores (post–pre difference) among the four groups.

**Figure 4:**
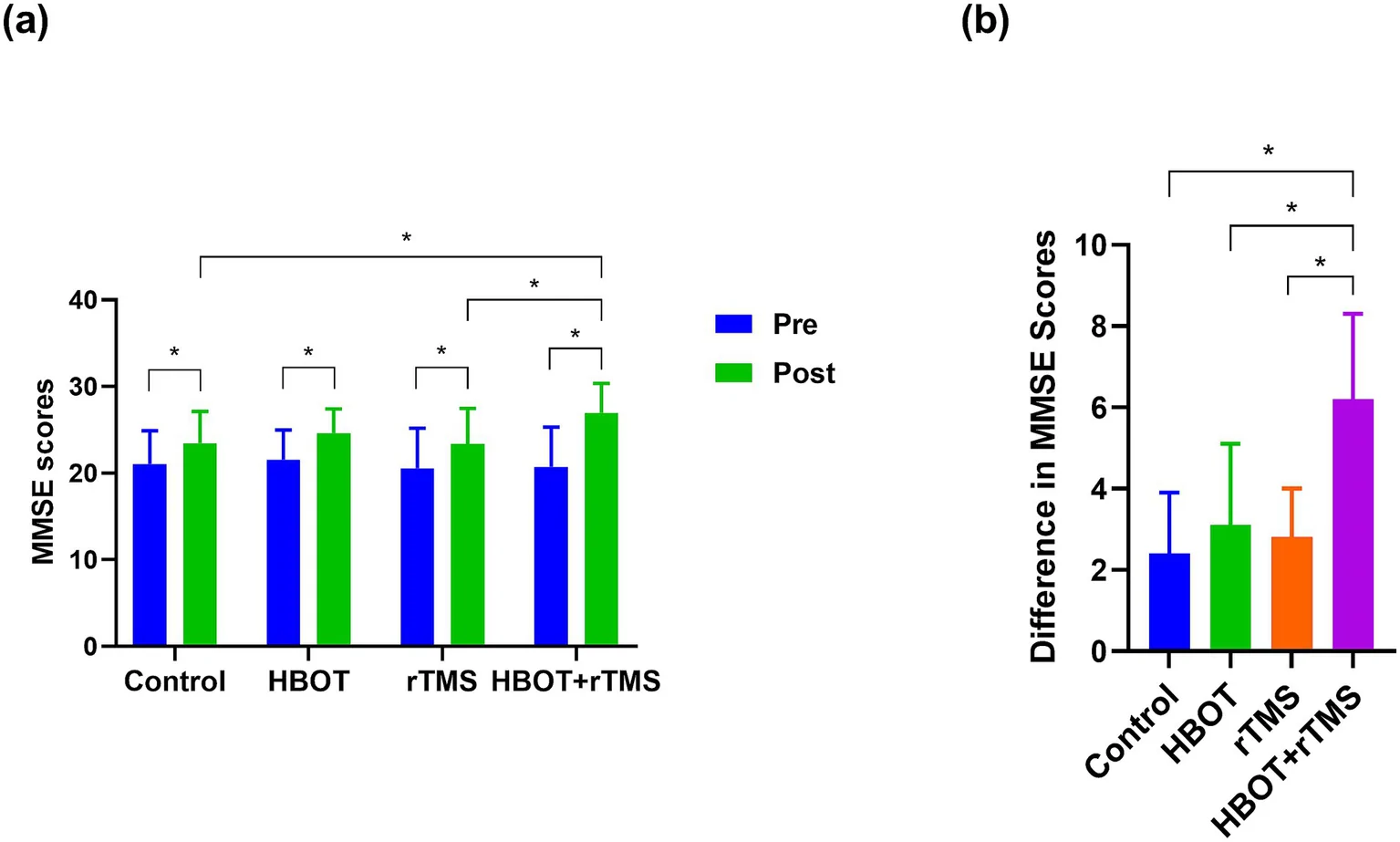
Comparison of MMSE scores before and after treatment in the four groups. **(a)** Within-group comparison of MMSE scores in each group. **(b)** Multiple comparisons of the change in total MMSE scores (post–pre difference) among the four groups.

No significant differences in MoCA or MMSE total scores were observed among the four groups at baseline (*p* > 0.05). Following the intervention, all groups showed significant within-group improvements from baseline in both MoCA and MMSE total scores (all *p* < 0.05) (Table 3, Figures 3A, 4A). There was a statistically significant difference in the magnitude of improvement (pre–post change) in both MoCA and MMSE scores among the four groups (all *p* < 0.05) (Table 3). *Post-hoc* multiple comparisons revealed that the improvement in MoCA or MMSE total scores for the combined HBOT and rTMS group was significantly greater than that in each of the other three groups (all *p* < 0.05) (Figures 3B, 4B).

**Table 3:** Comparison of MoCA, MMSE, MBI, SAS, and SDS scores before and after treatment among the four groups.

Variables		Control group (*n* = 18)	HBOT group (*n* = 18)	rTMS group (*n* = 18)	HBOT + rTMS group (*n* = 18)	*F*/*H*	*p*
MoCA	Pre-treatment	16.9 ± 4.9	16.9 ± 4.7	16.2 ± 4.6	16.4 ± 4.4	0.098	0.961
	Post-treatment	18.3 ± 5.3^a^	19.8 ± 4.8^a^	18.9 ± 4.4^a^	22.3 ± 4.6^a^	2.533	0.064
	Differences in MoCA scores	1.4 ± 1.9	2.9 ± 2.0	2.7 ± 2.1	5.9 ± 1.4	24.988	0.001
MMSE	Pre-treatment	21.1 ± 3.8	21.6 ± 3.4	20.6 ± 4.6	20.7 ± 4.6	0.204	0.893
	Post-treatment	23.4 ± 3.7^a^	24.6 ± 2.8^a^	23.3 ± 4.2^a^	26.9 ± 3.4^a^	4.022	0.011
	Differences in MMSE scores	2.4 ± 1.5	3.1 ± 2.0	2.8 ± 1.2	6.2 ± 2.1	18.499	0.001
MBI	Pre-treatment	54.3 ± 27.8	54.3 ± 24.8	53.7 ± 19.5	54.9 ± 21.3	0.007	0.999
	Post-treatment	61.3 ± 25.7^a^	67.7 ± 25.0^a^	64.2 ± 19.8^a^	74.2 ± 18.6^a^	1.097	0.357
	Differences in MBI scores	7.1 ± 6.1	13.3 ± 9.8	10.5 ± 9.6	19.3 ± 16.8	8.047	0.045
SAS	Pre-treatment	32 (12)	33.5 (7)	33 (16)	33 (10)	1.924	0.588
	Post-treatment	27.5 (11)^a^	30 (6)^a^	32.5 (8)^a^	28 (9)^a^	3.129	0.372
	Differences in SAS scores	−2 (3)	−4 (5)	−2.5 (9)	−5 (5)	4.956	0.175
SDS	Pre-treatment	38.2 ± 11.9	41.4 ± 9.6	39.2 ± 10.2	41.3 ± 9.0	0.441	0.725
	Post-treatment	33.9 ± 9.7^a^	35.9 ± 9.0 ^a^	34.7 ± 9.1^a^	33.2 ± 6.7^a^	0.323	0.809
	Differences in SDS scores	−4.3 ± 7.2	−5.5 ± 5.5	−4.4 ± 6.6	−8.1 ± 7.3	1.262	0.294

HBOT, hyperbaric oxygen therapy; rTMS, repetitive transcranial magnetic stimulation; MoCA, Montreal Cognitive Assessment; MMSE, Mini-Mental State Examination; MBI, modified barthel index; SAS, self-rating anxiety scale; SDS, Self-Rating Depression Scale. Data were presented as mean ± standard deviation for normally distributed variables and median (interquartile range) for non-normally distributed variables. Between-group comparisons before and after treatment were performed using one-way ANOVA or Kruskal–Wallis *H* test as appropriate. Within-group comparisons (pre- vs. post-treatment) were performed using paired *t*-test or Wilcoxon signed-rank test. The *F* and *H* values correspond to the respective test statistics.

a Significant in the within-group comparison at *p* < 0.05.

### 3.3 Activities of daily living

**Figure 5:**
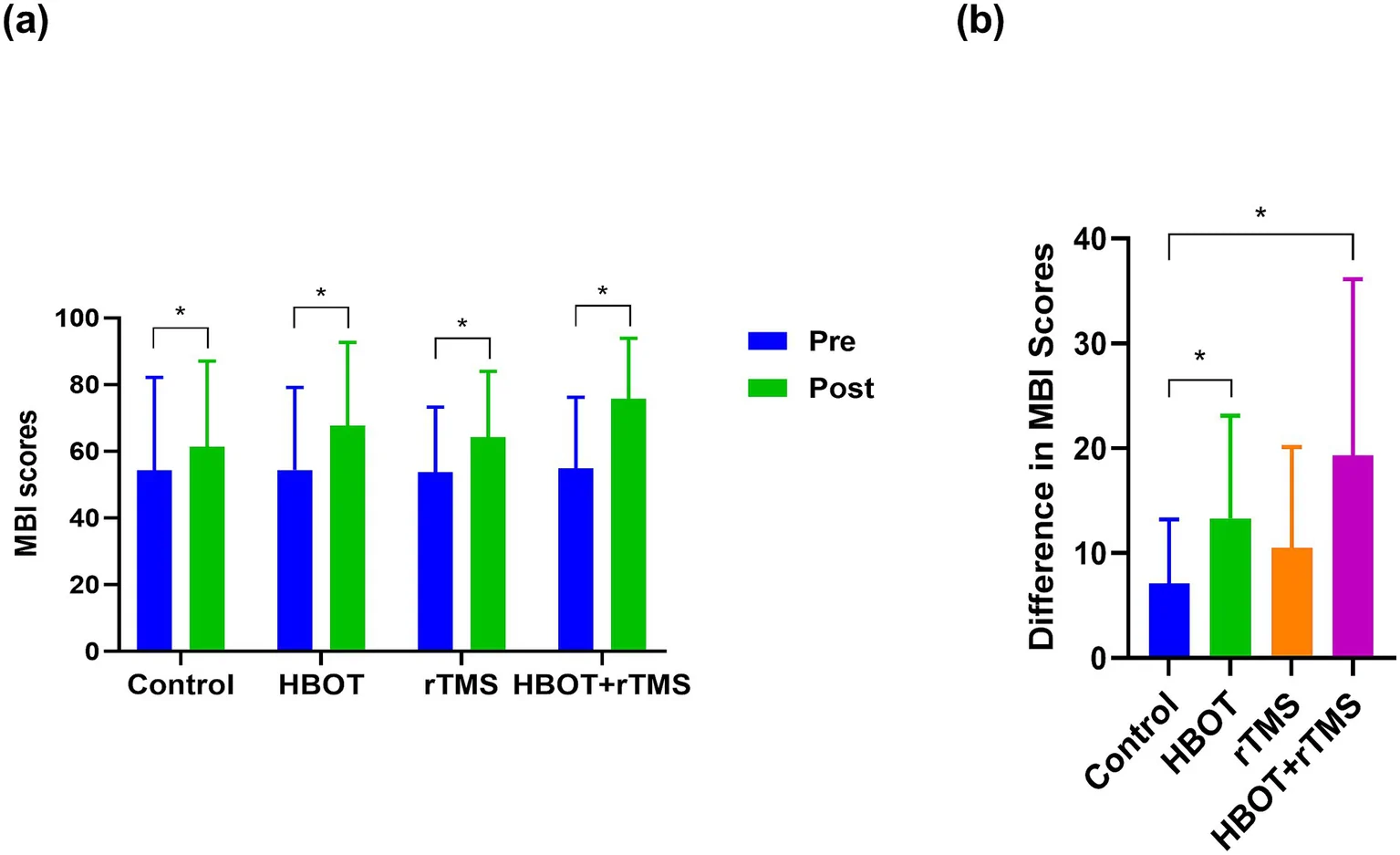
Comparison of MBI scores before and after treatment in the four groups. **(a)** Within-group comparison of MBI scores in each group. **(b)** Multiple comparisons of the change in total MBI scores (post–pre difference) among the four groups.

There was no significant difference in the total MBI score among the groups at the baseline (*p* > 0.05). Following the intervention, all groups demonstrated significant within-group improvements in MBI total scores from baseline (all *p* < 0.05) (Table 3, Figure 5A). A significant difference was found in the magnitude of improvement (pre–post change) in MBI scores among the four groups (*p* < 0.05) (Table 3). *Post-hoc* analysis indicated that the combined HBOT and rTMS group only showed a significantly greater increase in the MBI total score compared to the control group (*p* < 0.05) (Figure 5B). Subdomain analysis revealed that the combination group achieved significant within-group improvements in Grooming, dressing, toilet use, bathing, bed-chair transfer, ambulation, and stair climbing (all *p* < 0.05) (Table 4). Furthermore, its improvement in the ambulation was significantly greater than that in both the control group and rTMS group (all *p* < 0.05) (Table 4).

**Table 4:** Within-group and between-group comparisons of MBI domain scores among the four groups.

Variables	Control group (*n* = 18)		HBOT group (*n* = 18)		rTMS group (*n* = 18)		HBOT + rTMS group (*n* = 18)	
	Pre-treatment	Post-treatment	Pre-treatment	Post-treatment	Pre-treatment	Post-treatment	Pre-treatment	Post-treatment
Grooming	3 (3)	4 (2)^a^	3 (3)	5 (1)^a^	3 (3)	4 (2)^a^	4.5 (2)	5 (0)^a^
Feeding	10 (5)	10 (1)^a^	10 (3)	10 (1)	10 (5)	10 (2)^a^	10 (2)	10 (0)
Dressing	4 (6)	5 (8)	5 (7)	5 (8)	4 (3)	5 (4)^a^	5 (1)	8 (5)^a^
Toilet use	5 (7)	5 (7)^a^	5 (7)	6.5 (8)	5 (5)	5 (2)^a^	5 (7)	8 (5)^a^
Bathing	1 (3)	1 (2)^a^	1 (3)	1 (3)^a^	1 (3)	3 (2)^a^	1 (2)	3 (2)^a^
Bed-chair transfer	8 (15)	10 (12)^a^^,^^b^	8 (12)	12 (12)^a^	8 (13)	12 (8)^a^	3 (9)	13.5 (7)^a^
Ambulation	3 (12)	3.5 (12)^b^	3 (12)	10 (13)^a^	8 (13)	4 (12)^b^	3 (9)	8 (10)^a^
Stair climbing	0 (3)	1 (4)^a^	0 (3)	2 (6)^a^	1 (5)	0 (5)	0 (5)	5 (6)^a^
Bladder control	10 (0)	10 (0)	10 (0)	10 (0)	10 (0)	10 (0)	10 (0)	10 (0)
Bowel control	10 (0)	10 (0)	10 (0)	10 (0)	10 (0)	10 (0)	10 (0)	10 (0)

HBOT, hyperbaric oxygen therapy; rTMS, repetitive transcranial magnetic stimulation. Data were presented as median (interquartile range). Within-group comparisons were performed using Wilcoxon signed-rank test; between-group post-treatment comparisons were performed using Kruskal–Wallis *H* test with *post-hoc* pairwise comparisons (Dunn’s test).

a *p* < 0.05 for the within-group comparison.

b *p* < 0.05 for the comparison with the combination therapy group after treatment.

### 3.4 Anxiety and depression

No significant differences in SAS or SDS total scores were observed among the four groups at baseline (*p* > 0.05). After treatment, all groups showed significant within-group reductions from baseline in both SAS and SDS total scores (all *p* < 0.05) (Table 3). There was no significant difference was found in the magnitude of reduction (pre–post change) in both SAS and SDS scores among the four groups (all *p* > 0.05) (Table 3).

### 3.5 Cerebral cortex HbO₂ concentration

**Figure 6:**
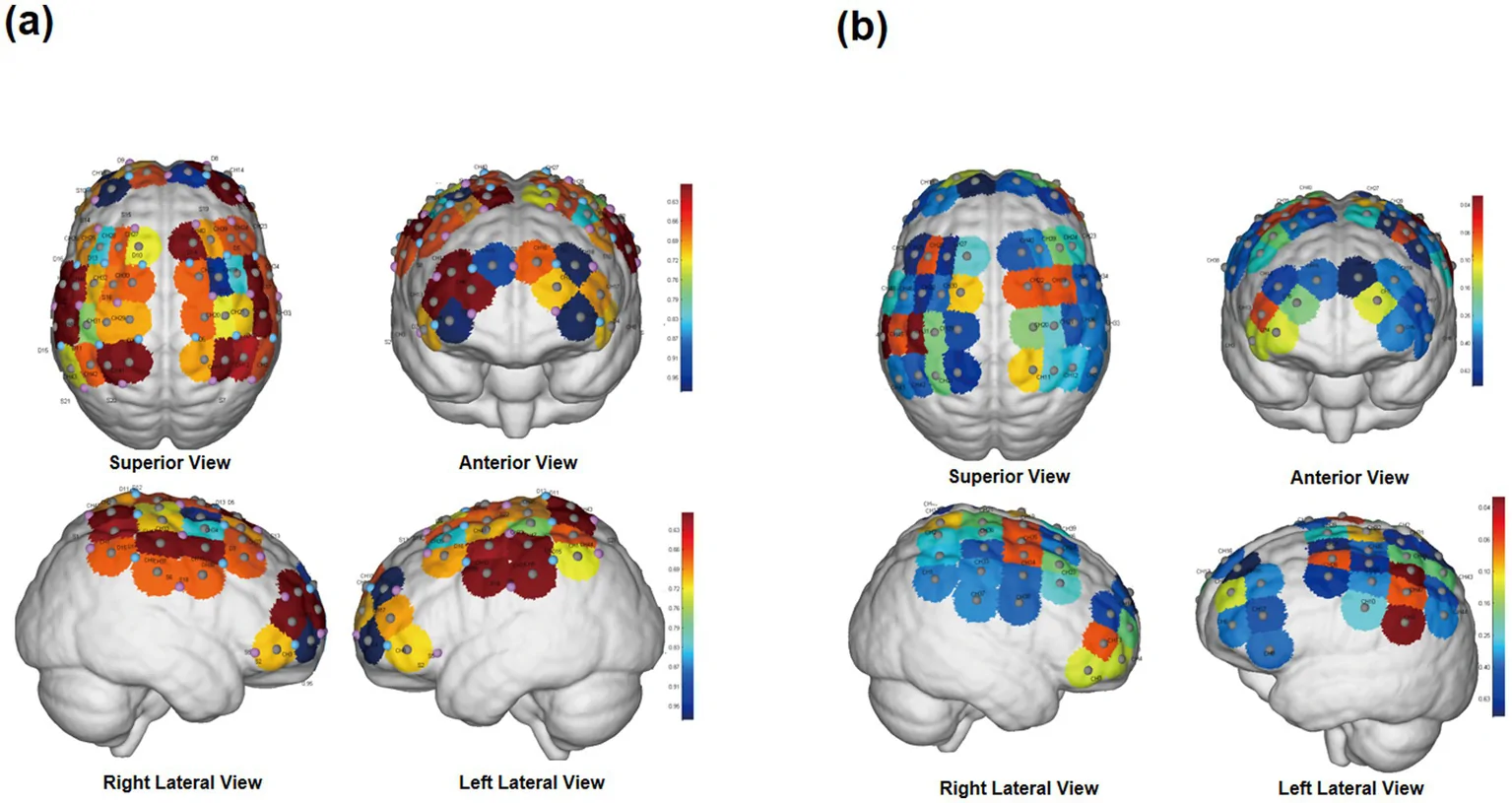
Comparison of HBO_2_ across 48 channels during the working memory task before and after treatment among the four groups. (a) Before treatment. (b) After treatment. The color bar scale shows FDR-corrected *P*-values, with red and blue representing higher and lower values, respectively.

Functional near-infrared spectroscopy (fNIRS) data were collected from 11 participants in each group. At baseline, no significant differences in HbO₂ concentration were observed across the 48 channels among the four groups (*p* > 0.05) (Supplementary Table S1, Figure 6). After treatment, significant differences in HbO₂ concentration were found in 13 channels (CH3, CH4, CH7, CH9, CH11, CH13, CH19, CH22, CH25, CH30, CH35, CH45, and CH47) among the four groups (all *p* < 0.05), while no significant differences were detected in the remaining 35 channels (all *p* > 0.05). After applying False Discovery Rate (FDR) correction for multiple comparisons, significant differences persisted in channels CH9 and CH45 (all *p* < 0.05), with no significant differences in the other 46 channels among the four groups (all *p* > 0.05) (Supplementary Table S1, Figure 6). To assess robustness against physiological noise, we performed a sensitivity analysis using ANCOVA with global signal regression (67). After controlling for baseline values and global components, the between-group differences were no longer significant for CH9 (*p* = 0.118) or CH45 (*p* = 0.073). No significant differences were found in the magnitude of pre- to post-treatment change in HbO₂ concentration across any of the 48 channels (all *p* > 0.05).

Spearman correlation analysis across the 44 participants with fNIRS data showed no significant correlations between the pre- to post-treatment changes (*Δ*) in CH9 and MoCA (rs = 0.14, *p* = 0.450), CH9 and MMSE (rs = 0.29, *p* = 0.104), CH45 and MoCA (rs = 0.16, *p* = 0.361), or CH45 and MMSE (rs = 0.28, *p* = 0.119).

### 3.6 Adverse events

One mild adverse event was reported during the trial. A participant in the rTMS group experienced a transient headache after the first session, which resolved with rest. This participant continued the study without further issues. All 72 participants completed the trial with no serious adverse events. The overall adverse event incidence was 1.39% (1/72), classified as mild (transient and self-resolving).

## 4 Discussion

This study explored the safety and efficacy of combined HBOT and rTMS in patients with VCI and employed fNIRS to monitor hemodynamic changes. Both HBOT and rTMS, administered alone or in combination, safely improved cognitive function, activities of daily living (ADL), and alleviated anxiety and depression. The combined therapy demonstrated superior cognitive benefits compared with either monotherapy or conventional treatment. However, the combined treatment only improved the patients’ ADL more than the conventional treatment, mainly in the aspect of ambulation, and did not yield additional benefits for emotional state. The findings suggested that HBOT or rTMS may enhance cognitive function by modulating the primary somatosensory and motor cortices in patients with VCI.

VCI can arise from various cerebrovascular pathologies, including large-artery atherosclerosis, cerebral small vessel disease, cardioembolism, and stroke (68). The onset of VCI was also associated with vascular risk factors, which may lead to cerebrovascular or neurological disorders, subsequently triggering cerebral hypoperfusion and dysfunction of neural networks (69, 70). Although the exact mechanisms remain incompletely understood, chronic, age-related dysregulation of cerebral blood flow (CBF) represented a common underlying pathological basis (69, 70). Additional contributors included chronic hypoxia, increased blood–brain barrier permeability, endothelial dysfunction, systemic inflammation, and the ensuing activation of intracerebral innate immune responses (69, 70). Proper regulation of CBF was crucial for maintaining stable cerebral perfusion, blood volume, and intracranial pressure, thereby preventing high-pressure flow-induced damage to the distal microvascular system (71). Chronic dysregulation of CBF can lead to white matter injury, cerebral atrophy, stroke, and memory impairment (72). The normal regulation of CBF was highly dependent on the function of the neurovascular unit (NVU). Risk factors such as hypertension and aging can cause vascular endothelial injury, disrupt blood–brain barrier integrity, lead to neurovascular dysfunction, and promote microvascular thrombosis, thereby impairing the normal regulation of CBF (39). NVU dysfunction may thus contribute to VCI progression by impairing neurovascular coupling and compromising brain function during cognitive tasks (40).

Our findings demonstrated that combined HBOT and rTMS treatment significantly enhanced cognitive function in patients with VCI. A meta-analysis by Zhou et al. (65) confirmed that rTMS significantly improved MoCA scores in patients with VCI, and Lagoda et al. (41) recently reported that high-frequency rTMS over the left DLPFC improved cognitive function in cerebral small vessel disease, a major VCI subtype. For combination therapy, Huang et al. (42) demonstrated that combined HBOT and rTMS significantly improved cognitive function and ADL in patients with non-dementia VCI, while Zhang et al. (43) showed that HBOT augmented the cognitive benefits of cognitive training in VCI. However, regarding the improvement of ADL, the combined therapy in this study did not confer additional benefit compared to monotherapy, which differs from the findings of Huang et al. (42) and may be attributable to the shorter treatment duration. Similarly, we observed no significantly added improvement in anxiety or depression, compared to monotherapy or conventional treatment. Previous combination therapy studies on emotional outcomes have predominantly focused on post-stroke populations, and VCI-specific evidence in this domain remains an important area for future investigation. Notably, the cognitive improvements were unlikely to be driven merely by mood changes, as there were no significant between-group differences in SAS or SDS improvements (*p* > 0.05), whereas the combined group showed significantly greater MoCA gains (*p* < 0.05). The discrepancies between our results and prior VCI studies may be explained by differences in sample characteristics, disease severity, and treatment length. The combined HBOT and rTMS regimen in this study demonstrated a favorable safety profile, only one mild, reversible adverse event (transient headache after rTMS) occurred, and no serious adverse events were recorded. This aligned with existing safety data: rTMS was well-tolerated as a non-invasive intervention, and a large-scale retrospective study reported an HBOT adverse event only 0.68% (44).

HBOT and rTMS exerted neuroprotective and neuromodulatory effects through distinct yet complementary mechanisms targeting cognitive impairment. Hypoxia was a key pathological factor in neuronal injury. HBOT improved the cerebral microenvironment by increasing arterial oxygen partial pressure, enhancing tissue oxygenation, reducing intracranial pressure, and alleviating cerebral edema. Basic research suggested HBOT downregulated inflammatory mediators like COX-2 (45), inhibited apoptosis-related factors such as caspase-3 activity, and enhanced antioxidant defenses (46). A meta-analysis of animal models further supported that HBOT reduced cerebral edema and improved neurobehavioral outcomes (47). Collectively, HBOT may improve cognitive function through multiple pathways: enhancing oxygenation, promoting angiogenesis, reducing inflammation and oxidative stress, maintaining mitochondrial function, and supporting cellular repair and regeneration (6). rTMS focused on non-invasive neuromodulation of specific brain regions. Studies on cognitive improvement often targeted the left dorsolateral prefrontal cortex (DLPFC)—a region involved in processing speed, attention, working memory, and episodic memory (48), and a key node of the central executive network (49). A meta-analysis indicated that high-frequency rTMS over the left DLPFC could enhance cognitive performance in various populations, including those with Alzheimer’s disease and depression (31). It was generally accepted that stimulation at 10–20 Hz within 80–110% of the motor threshold most effectively induced cognitive improvement (31). The potential mechanisms of rTMS for cognitive enhancement involved promoting long-term potentiation, enhancing neural and synaptic plasticity, upregulating brain-derived neurotrophic factor and vascular endothelial growth factor, and modulating NMDA receptor function and hippocampal neurogenesis (50). The complementarity of HBOT and rTMS lies in their targeting of different pathological pathways that converge on neurovascular repair. HBOT primarily improves cerebral oxygenation and reduces neuroinflammation, whereas rTMS directly enhances synaptic plasticity and cortical excitability. These mechanisms may converge through shared upregulation of BDNF and VEGF (12, 51), and HBOT-induced oxygenation may potentiate rTMS-induced neuroplasticity (52). Together, the combined regimen may produce synergistic effects by simultaneously restoring microvascular function and neural circuit integrity. Future studies are needed to investigate these shared mechanisms and determine the optimal treatment sequence.

Our fNIRS data revealed that, after different interventions, significant between-group differences in HbO₂ concentration during a working memory task in VCI patients (FDR-corrected *p* < 0.05) were localized to channels CH9 and CH45. Working memory tasks typically involved the coordinated activity of multiple brain regions, including the prefrontal, sensory, and posterior parietal cortices. Based on Brodmann area mapping, CH9 primarily covered the primary somatosensory cortex (S1) and the supramarginal gyrus of Wernicke’s area, while CH45 covered both S1 and the primary motor cortex (M1). The supramarginal gyrus was involved in verbal fluency. S1, located in the postcentral gyrus and functionally connected to the thalamus, not only processed basic somatosensory information but was also widely engaged in higher cognitive processes such as learning, memory, emotion recognition, and body representation (53–55), and played a significant role in the cognitive control of language (56). Notably, S1 could be specifically recruited during working memory tasks that involve body related information, suggesting the existence of a mnemonic system dedicated to bodily information where S1 played a key role due to its visuo-tactile cross-modal integration properties (54). Galvez-Pol et al. (57) further demonstrated that both S1 and the motor cortex were activated during visual working memory tasks requiring the maintenance of bodily information. Moreover, 10 Hz rTMS could enhance behavioral performance in visual working memory tasks, a mechanism possibly related to increased visuo-tactile response synchronization within S1 and its entraining to alpha-band frequencies (58).

Collectively, this evidence indicated that S1 was a key brain region with cross-modal integration, bridging sensory processing and cognition. Although M1 was traditionally considered primarily responsible for motor execution, recent studies implicated its involvement in higher cognitive processes including attention, motor learning, and inhibitory control (60). Therefore, differences in channels CH9 and CH45 post-treatment suggested that HBOT or rTMS might promote cognitive function by modulating S1 and M1 in patients with VCI. The absence of significant between-group differences in the prefrontal and posterior parietal cortices might have been due to the limited sample size. Correlation analyses did not reveal significant associations between the HbO₂ changes in CH9/CH45 and cognitive improvements. This may be due to the limited sample size of fNIRS data (*n* = 44), the etiological heterogeneity of VCI, or the possibility that the cognitive benefits of HBOT and rTMS are mediated by a more widely distributed cortical network rather than localized activation in S1 and M1 alone.

Task-based fNIRS studies examining HBOT-induced hemodynamics changes in cognitive impairment were scarce, and its application in rTMS cognitive research remained limited. A recent systematic review has comprehensively summarized the utility of fNIRS in stroke rehabilitation for monitoring brain function and evaluating interventional responses (61). Liu et al. (62) found that high-frequency rTMS enhanced prefrontal-sensorimotor network activation during Stroop tasks in post-stroke patients with executive dysfunction. Curtin et al. (73) reported that rTMS improved the neural efficiency of the left DLPFC in healthy participants during a symbol-digit substitution task. Yamanaka et al. (74) further revealed that the improvement in spatial working memory tasks with real rTMS in healthy individuals was associated with activation of the right parietal cortex and increased frontal HbO₂ concentration. They also observed compensatory over-recruitment of HbO₂ in older adults during working memory tasks (75). These results indicated that task-based fNIRS could sensitively capture brain functional changes induced by neuromodulation and held promise as an effective tool for assessing treatment response.

This study has several limitations. First, blinding of patients and intervenors was not implemented. Second, subgroup analysis based on lesion location was not performed. Third, the intervention period was relatively short (3 weeks) with no long-term follow-up. Fourth, the sample size was small. Fifth, the VCI cases in this study were limited to those caused by ischemic and hemorrhagic stroke, and patients with other VCI subtypes such as isolated cerebral small vessel disease were not included, which may limit the generalizability of our findings. Sixth, our sensitivity analysis showed that the significance of CH9 and CH45 did not persist after global signal correction (*p* > 0.05), indicating that physiological noise may have contributed. Future studies should incorporate short-distance channels or more advanced correction methods. Seventh, we did not administer standardized stroke severity scales (e.g., NIHSS or FMA), which limits detailed sample characterization. However, the four groups were comparable in lesion type, lesion side, disease duration, and baseline cognitive performance (Table 2). Finally, the specific fNIRS analytical details were not preregistered. Preregistration of fNIRS analytical protocols is recommended to enhance transparency and reproducibility (76). Future studies with larger samples, longer durations, short-distance channels, and incorporating lesion stratification and long-term follow-up are needed to further validate these conclusions. Another promising direction is the integration of rTMS with concurrent cognitive tasks or closed-loop neurofeedback approaches, which may further enhance cognitive benefits (77).

In conclusion, this study confirmed that a 3-week combined regimen of HBOT and rTMS is safe and effective in improving cognitive function in VCI patients, with efficacy superior to monotherapy. This cognitive benefit may involve modulating neural activity in S1 and M1. However, the combined regimen did not yield greater improvement in daily living abilities or emotional state compared to either therapy alone. Larger, longer-term, and methodologically rigorous studies are warranted to confirm the long-term efficacy and mechanisms of this combined intervention.

## Data Availability

The original contributions presented in the study are included in the article/Supplementary material, further inquiries can be directed to the corresponding author.
