# Supplementary material for: Combined hyperbaric oxygen and repetitive transcranial magnetic stimulation for vascular cognitive impairment: a randomized controlled trail
Source: Front Neurol. 2026 Aug 18;17:1826010. doi: 10.3389/fneur.2026.1826010 (PMC13529544; doi:10.3389/fneur.2026.1826010)
Supplement: Supplementary file 1 [file Table_1.docx]

**Supplementary Table S1. Comparison of HBO_2_ across 48 channels during the working memory task before and after treatment among the four groups.**

|  |  | **Control group（n=18）** | **HBOT group（n=18）** | **rTMS group（n=18）** | **HBOT + rTMS group（n=18）** | ***F*** | ***P*** | ***P* (FDR-corrected)** |
| --- | --- | --- | --- | --- | --- | --- | --- | --- |
| **CH1 (S1-D1)** | **Pre-treatment** | 0.001±0.036 | 0.017±0.041 | 0.026±0.040 | 0.034±0.057 | 1.123 | 0.351 | 0.674 |
|  | **Post-treatment** | 0.019±0.026 | 0.011±0.066 | 0.027±0.025 | 0.048±0.041 | 1.509 | 0.227 | 0.355 |
| **CH2 (S1-D12)** | **Pre-treatment** | 0.006±0.043 | -0.001±0.032 | 0.019±0.044 | 0.035±0.041 | 1.704 | 0.182 | 0.623 |
|  | **Post-treatment** | 0.024±0.032 | 0.005±0.061 | 0.022±0.039 | 0.054±0.056 | 1.920 | 0.142 | 0.284 |
| **CH3 (S2-D2)** | **Pre-treatment** | 0.023±0.048 | 0.020±0.061 | 0.012±0.138 | 0.066±0.081 | 0.794 | 0.504 | 0.707 |
|  | **Post-treatment** | 0.037±0.068 | 0.028±0.042 | 0.024±0.036 | 0.080±0.036 | 3.284 | 0.030 | 0.126 |
| **CH4 (S3-D2)** | **Pre-treatment** | 0.047±0.059 | 0.052±0.047 | 0.046±0.071 | 0.061±0.098 | 0.098 | 0.960 | 0.981 |
|  | **Post-treatment** | 0.045±0.045 | 0.022±0.053 | 0.034±0.049 | 0.091±0.074 | 3.184 | 0.034 | 0.126 |
| **CH5 (S3-D8)** | **Pre-treatment** | 0.016±0.038 | 0.018±0.044 | 0.021±0.038 | 0.053±0.039 | 2.124 | 0.112 | 0.606 |
|  | **Post-treatment** | 0.028±0.033 | 0.014±0.028 | 0.005±0.038 | 0.048±0.049 | 2.658 | 0.061 | 0.176 |
| **CH6 (S4-D4)** | **Pre-treatment** | 0.049±0.052 | 0.045±0.054 | 0.041±0.054 | 0.048±0.065 | 0.044 | 0.987 | 0.987 |
|  | **Post-treatment** | 0.037±0.062 | 0.012±0.032 | 0.020±0.079 | 0.060±0.050 | 1.472 | 0.237 | 0.355 |
| **CH7 (S4-D9)** | **Pre-treatment** | 0.007±0.031 | 0.015±0.060 | 0.029±0.046 | 0.039±0.071 | 0.779 | 0.513 | 0.707 |
|  | **Post-treatment** | 0.034±0.037 | 0.000±0.028 | 0.010±0.046 | 0.044±0.038 | 3.211 | 0.033 | 0.126 |
| **CH8 (S5-D4)** | **Pre-treatment** | 0.049±0.060 | 0.020±0.030 | 0.017±0.078 | 0.057±0.123 | 0.711 | 0.551 | 0.715 |
|  | **Post-treatment** | 0.032±0.086 | 0.040±0.043 | 0.001±0.036 | 0.050±0.060 | 1.350 | 0.272 | 0.384 |
| **CH9 (S6-D15)** | **Pre-treatment** | 0.031±0.040 | -0.007±0.033 | 0.043±0.062 | 0.041±0.061 | 2.334 | 0.088 | 0.606 |
|  | **Post-treatment** | 0.032±0.019 | 0.005±0.048 | 0.003±0.023 | 0.071±0.061 | 6.545 | 0.001 | 0.034 |
| **CH10 (S6-D16)** | **Pre-treatment** | 0.032±0.033 | 0.008±0.021 | 0.008±0.033 | 0.038±0.053 | 1.995 | 0.130 | 0.606 |
|  | **Post-treatment** | 0.011±0.059 | 0.016±0.050 | 0.002±0.052 | 0.061±0.075 | 2.123 | 0.112 | 0.245 |
| **CH11 (S7-D6)** | **Pre-treatment** | 0.012±0.031 | -0.003±0.032 | 0.015±0.017 | 0.014±0.037 | 0.902 | 0.449 | 0.699 |
|  | **Post-treatment** | 0.025±0.028 | -0.002±0.042 | 0.004±0.027 | 0.045±0.047 | 3.654 | 0.020 | 0.097 |
| **CH12 (S7-D12)** | **Pre-treatment** | 0.006±0.035 | -0.023±0.046 | 0.022±0.018 | 0.016±0.055 | 2.636 | 0.063 | 0.606 |
|  | **Post-treatment** | 0.015±0.037 | 0.009±0.035 | 0.005±0.033 | 0.039±0.039 | 2.032 | 0.125 | 0.260 |
| **CH13 (S8-D2)** | **Pre-treatment** | 0.022±0.049 | 0.015±0.067 | 0.022±0.086 | 0.081±0.084 | 1.932 | 0.140 | 0.606 |
|  | **Post-treatment** | 0.037±0.035 | 0.027±0.048 | 0.034±0.040 | 0.089±0.057 | 4.281 | 0.010 | 0.066 |
| **CH14 (S8-D8)** | **Pre-treatment** | 0.031±0.038 | 0.016±0.038 | 0.017±0.043 | 0.058±0.058 | 2.055 | 0.122 | 0.606 |
|  | **Post-treatment** | 0.021±0.051 | 0.028±0.032 | 0.020±0.024 | 0.047±0.056 | 0.976 | 0.414 | 0.473 |
| **CH15 (S9-D8)** | **Pre-treatment** | 0.018±0.033 | 0.023±0.078 | 0.005±0.024 | 0.020±0.045 | 0.282 | 0.838 | 0.915 |
|  | **Post-treatment** | 0.014±0.052 | 0.008±0.025 | 0.004±0.022 | 0.031±0.032 | 1.304 | 0.287 | 0.393 |
| **CH16 (S9-D9)** | **Pre-treatment** | -0.003±0.040 | 0.025±0.039 | 0.015±0.036 | 0.036±0.067 | 1.315 | 0.283 | 0.674 |
|  | **Post-treatment** | 0.010±0.043 | 0.010±0.043 | 0.014±0.029 | 0.024±0.040 | 0.310 | 0.818 | 0.818 |
| **CH17 (S10-D4)** | **Pre-treatment** | 0.033±0.038 | 0.011±0.033 | 0.046±0.077 | 0.044±0.050 | 1.012 | 0.397 | 0.699 |
|  | **Post-treatment** | 0.023±0.055 | 0.022±0.035 | 0.019±0.028 | 0.049±0.055 | 1.065 | 0.375 | 0.439 |
| **CH18 (S10-D9)** | **Pre-treatment** | 0.019±0.029 | 0.031±0.053 | 0.027±0.086 | 0.034±0.064 | 0.119 | 0.948 | 0.981 |
|  | **Post-treatment** | 0.006±0.048 | 0.012±0.035 | 0.021±0.025 | 0.040±0.040 | 1.695 | 0.375 | 0.344 |
| **CH19 (S12-D5)** | **Pre-treatment** | 0.011±0.037 | 0.020±0.034 | 0.010±0.037 | 0.018±0.049 | 0.190 | 0.903 | 0.963 |
|  | **Post-treatment** | 0.036±0.026 | -0.002±0.031 | 0.002±0.025 | 0.022±0.026 | 4.819 | 0.006 | 0.066 |
| **CH20 (S12-D6)** | **Pre-treatment** | 0.010±0.031 | 0.021±0.038 | 0.001±0.037 | 0.027±0.035 | 1.248 | 0.305 | 0.674 |
|  | **Post-treatment** | 0.031±0.023 | 0.002±0.029 | -0.001±0.044 | 0.025±0.029 | 2.783 | 0.053 | 0.170 |
| **CH21 (S12-D12)** | **Pre-treatment** | 0.017±0.034 | 0.006±0.024 | 0.008±0.033 | 0.025±0.048 | 0.615 | 0.609 | 0.731 |
|  | **Post-treatment** | 0.045±0.032 | 0.011±0.031 | 0.010±0.034 | 0.029±0.048 | 2.236 | 0.099 | 0.242 |
| **CH22 (S12-D14)** | **Pre-treatment** | 0.012±0.033 | 0.004±0.031 | 0.028±0.033 | 0.008±0.027 | 1.238 | 0.309 | 0.674 |
|  | **Post-treatment** | 0.037±0.026 | 0.004±0.037 | -0.010±0.035 | 0.030±0.037 | 4.496 | 0.008 | 0.066 |
| **CH23 (S13-D3)** | **Pre-treatment** | 0.016±0.030 | 0.026±0.046 | 0.014±0.062 | 0.049±0.054 | 1.184 | 0.328 | 0.674 |
|  | **Post-treatment** | 0.029±0.057 | 0.007±0.057 | 0.023±0.042 | 0.066±0.065 | 2.176 | 0.106 | 0.242 |
| **CH24 (S13-D5)** | **Pre-treatment** | 0.017±0.030 | 0.008±0.027 | 0.030±0.034 | 0.035±0.053 | 1.134 | 0.347 | 0.674 |
|  | **Post-treatment** | 0.036±0.037 | -0.001±0.045 | 0.011±0.030 | 0.043±0.050 | 2.841 | 0.050 | 0.170 |
| **CH25 (S14-D13)** | **Pre-treatment** | 0.016±0.040 | 0.014±0.047 | 0.028±0.043 | 0.035±0.066 | 0.418 | 0.741 | 0.827 |
|  | **Post-treatment** | 0.016±0.044 | 0.002±0.034 | 0.003±0.022 | 0.053±0.041 | 4.712 | 0.007 | 0.066 |
| **CH26 (S14-D16)** | **Pre-treatment** | -0.007±0.047 | 0.007±0.066 | 0.019±0.025 | 0.029±0.068 | 0.909 | 0.445 | 0.699 |
|  | **Post-treatment** | 0.015±0.048 | 0.007±0.038 | 0.013±0.038 | 0.030±0.041 | 0.623 | 0.604 | 0.655 |
| **CH27 (S15-D10)** | **Pre-treatment** | 0.018±0.041 | 0.004±0.024 | 0.022±0.011 | 0.020±0.047 | 0.640 | 0.594 | 0.731 |
|  | **Post-treatment** | 0.018±0.031 | -0.004±0.021 | 0.008±0.027 | 0.020±0.026 | 2.208 | 0.102 | 0.242 |
| **CH28 (S15-D13)** | **Pre-treatment** | 0.027±0.051 | 0.012±0.019 | 0.009±0.015 | 0.038±0.049 | 1.420 | 0.251 | 0.674 |
|  | **Post-treatment** | 0.016±0.033 | 0.006±0.039 | 0.018±0.027 | 0.025±0.043 | 0.516 | 0.674 | 0.688 |
| **CH29 (S16-D7)** | **Pre-treatment** | 0.018±0.041 | -0.022±0.124 | -0.020±0.094 | 0.021±0.034 | 0.896 | 0.451 | 0.699 |
|  | **Post-treatment** | 0.043±0.036 | 0.096±0.249 | 0.011±0.018 | 0.029±0.033 | 0.919 | 0.440 | 0.492 |
| **CH30 (S16-D10)** | **Pre-treatment** | 0.006±0.037 | 0.045±0.082 | 0.022±0.036 | 0.031±0.028 | 1.142 | 0.344 | 0.674 |
|  | **Post-treatment** | 0.021±0.027 | -0.002±0.027 | 0.008±0.033 | 0.037±0.028 | 3.772 | 0.018 | 0.095 |
| **CH31 (S16-D11)** | **Pre-treatment** | 0.018±0.043 | 0.006±0.041 | 0.014±0.034 | 0.031±0.024 | 0.958 | 0.422 | 0.699 |
|  | **Post-treatment** | 0.046±0.034 | 0.008±0.040 | 0.019±0.037 | 0.040±0.035 | 2.590 | 0.066 | 0.176 |
| **CH32 (S16-D13)** | **Pre-treatment** | 0.019±0.029 | -0.002±0.055 | 0.010±0.036 | 0.026±0.034 | 1.079 | 0.369 | 0.681 |
|  | **Post-treatment** | 0.017±0.036 | -0.002±0.026 | 0.006±0.028 | 0.024±0.040 | 1.363 | 0.268 | 0.384 |
| **CH33 (S17-D1)** | **Pre-treatment** | 0.008±0.030 | 0.001±0.034 | 0.001±0.025 | 0.036±0.039 | 2.920 | 0.046 | 0.606 |
|  | **Post-treatment** | 0.023±0.042 | 0.002±0.061 | 0.042±0.042 | 0.042±0.047 | 1.666 | 0.190 | 0.344 |
| **CH34 (S17-D3)** | **Pre-treatment** | 0.024±0.032 | -0.005±0.052 | -0.003±0.038 | 0.032±0.052 | 2.002 | 0.129 | 0.606 |
|  | **Post-treatment** | 0.034±0.036 | 0.009±0.040 | 0.027±0.039 | 0.042±0.051 | 1.248 | 0.305 | 0.396 |
| **CH35 (S17-D5)** | **Pre-treatment** | 0.020±0.035 | 0.030±0.045 | 0.019±0.037 | 0.038±0.062 | 0.444 | 0.723 | 0.826 |
|  | **Post-treatment** | 0.044±0.032 | -0.004±0.032 | 0.011±0.028 | 0.032±0.044 | 4.219 | 0.011 | 0.066 |
| **CH36 (S17-D12)** | **Pre-treatment** | 0.028±0.041 | 0.009±0.022 | 0.016±0.021 | 0.026±0.045 | 0.739 | 0.535 | 0.713 |
|  | **Post-treatment** | 0.038±0.042 | 0.006±0.045 | 0.032±0.061 | 0.040±0.045 | 1.148 | 0.342 | 0.420 |
| **CH37 (S18-D1)** | **Pre-treatment** | 0.007±0.057 | 0.015±0.039 | -0.008±0.057 | 0.037±0.040 | 1.564 | 0.213 | 0.674 |
|  | **Post-treatment** | 0.036±0.041 | 0.007±0.051 | 0.028±0.048 | 0.050±0.055 | 1.479 | 0.235 | 0.355 |
| **CH38 (S18-D3)** | **Pre-treatment** | 0.011±0.052 | 0.012±0.038 | -0.000±0.069 | 0.043±0.058 | 1.209 | 0.319 | 0.674 |
|  | **Post-treatment** | 0.031±0.033 | 0.009±0.043 | 0.006±0.060 | 0.047±0.083 | 1.250 | 0.304 | 0.396 |
| **CH39 (S19-D5)** | **Pre-treatment** | 0.016±0.030 | 0.013±0.015 | 0.024±0.045 | 0.034±0.039 | 0.868 | 0.466 | 0.699 |
|  | **Post-treatment** | 0.031±0.041 | 0.003±0.025 | 0.001±0.049 | 0.023±0.038 | 1.586 | 0.208 | 0.344 |
| **CH40 (S19-D14)** | **Pre-treatment** | 0.011±0.019 | 0.004±0.020 | 0.027±0.034 | 0.020±0.023 | 1.862 | 0.151 | 0.606 |
|  | **Post-treatment** | 0.011±0.033 | -0.001±0.025 | 0.007±0.060 | 0.028±0.027 | 1.122 | 0.352 | 0.422 |
| **CH41 (S20-D7)** | **Pre-treatment** | 0.011±0.026 | -0.037±0.102 | -0.001±0.032 | 0.027±0.045 | 2.322 | 0.090 | 0.606 |
|  | **Post-treatment** | 0.029±0.023 | 0.074±0.235 | 0.012±0.029 | 0.019±0.018 | 0.608 | 0.614 | 0.655 |
| **CH42 (S20-D11)** | **Pre-treatment** | 0.016±0.019 | -0.008±0.039 | 0.008±0.025 | 0.031±0.030 | 3.387 | 0.027 | 0.606 |
|  | **Post-treatment** | 0.032±0.019 | 0.018±0.048 | -0.003±0.035 | 0.032±0.028 | 2.592 | 0.066 | 0.176 |
| **CH43 (S21-D11)** | **Pre-treatment** | 0.010±0.057 | 0.012±0.023 | 0.022±0.029 | 0.039±0.034 | 1.398 | 0.257 | 0.674 |
|  | **Post-treatment** | 0.040±0.020 | 0.017±0.047 | 0.011±0.036 | 0.030±0.050 | 1.171 | 0.333 | 0.420 |
| **CH44 (S21-D15)** | **Pre-treatment** | 0.016±0.033 | 0.016±0.049 | 0.020±0.042 | 0.038±0.047 | 0.624 | 0.603 | 0.731 |
|  | **Post-treatment** | 0.031±0.017 | 0.005±0.052 | 0.004±0.033 | 0.032±0.051 | 1.585 | 0.208 | 0.344 |
| **CH45 (S22-D11)** | **Pre-treatment** | 0.021±0.043 | 0.022±0.043 | 0.011±0.030 | 0.034±0.052 | 0.542 | 0.656 | 0.768 |
|  | **Post-treatment** | 0.045±0.043 | 0.010±0.027 | -0.010±0.043 | 0.054±0.044 | 6.224 | 0.001 | 0.034 |
| **CH46 (S22-D13)** | **Pre-treatment** | 0.020±0.025 | 0.001±0.072 | 0.007±0.031 | 0.027±0.038 | 0.773 | 0.516 | 0.707 |
|  | **Post-treatment** | 0.015±0.040 | 0.003±0.035 | 0.007±0.033 | 0.022±0.044 | 0.579 | 0.632 | 0.660 |
| **CH47 (S22-D15)** | **Pre-treatment** | 0.014±0.042 | 0.006±0.034 | 0.015±0.031 | 0.047±0.054 | 2.175 | 0.106 | 0.606 |
|  | **Post-treatment** | 0.036±0.029 | 0.002±0.039 | 0.002±0.036 | 0.061±0.067 | 4.379 | 0.009 | 0.066 |
| **CH48 (S22-D16)** | **Pre-treatment** | 0.000±0.052 | 0.007±0.034 | 0.011±0.020 | 0.036±0.041 | 1.775 | 0.167 | 0.618 |
|  | **Post-treatment** | 0.010±0.040 | 0.008±0.040 | 0.007±0.032 | 0.039±0.050 | 1.586 | 0.208 | 0.344 |

Abbreviations: HBO_2_, oxygenated hemoglobin; HBOT, hyperbaric oxygen therapy; rTMS, repetitive transcranial magnetic stimulation; FDR, false discovery rate; CH, channel; S, source; D, detector. Data were presented as mean ± standard deviation. The F and P values were obtained from one-way ANOVA across the four groups. P (FDR-corrected) values were calculated using the Benjamini-Hochberg false discovery rate correction for multiple comparisons across 48 channels.
